# Supplementary material for: Mapping the evolution of fertility support policies in China: A content and instrumental analysis
Source: PLoS One. 2025 Oct 9;20(10):e0332137. doi: 10.1371/journal.pone.0332137 (PMC12510515; doi:10.1371/journal.pone.0332137)
Supplement: S1 Appendix — (ZIP) [file pone.0332137.s001.zip › S1 Appendix. 226 original policy documents/218-第八届全国人民代表大会第四次会议关于国民经济和社会发展“九五”计划和2010年远景目标纲要及关于《纲要》报告的决议(FBM-CLI-1-320340).doc]

第八届全国人民代表大会第四次会议关于国民经济和社会发展“九五”计划和2010年远景目标纲要及关于《纲要》报告的决议

**发布部门：** [全国人民代表大会](https://www.pkulaw.com/law/chl?Aggs.IssueDepartment=101)

**发布日期：**1996.03.17

**实施日期：**1996.03.17

**时效性：** [现行有效](https://www.pkulaw.com/law/chl?Aggs.TimelinessDic=01)

**效力级别：** [工作文件](https://www.pkulaw.com/law/chl?Aggs.EffectivenessDic=XA0105)

**法规类别：** [人大议事](https://www.pkulaw.com/law/chl?Aggs.Category=00106)

**专题分类：** [依法治国](https://www.pkulaw.com/law/chl?Aggs.SpecialType=019)

第八届全国人民代表大会第四次会议关于国民经济和社会发展“九五”计划和2010年远景目标纲要及关于《纲要》报告的决议
（1996年3月17日第八届全国人民代表大会第四次会议通过）

　　第八届全国人民代表大会第四次会议，讨论和审查了国务院提出的《国民经济和社会发展“九五”计划和2010年远景目标纲要（草案）》，审议了李鹏总理代表国务院所作的《关于国民经济和社会发展“九五”计划和2010年远景目标纲要的报告》。会议认为，《纲要（草案）》和报告提出的今后十五年的奋斗目标和指导方针，国民经济和社会发展的主要任务和战略布局，深化经济体制改革和扩大对外开放的部署和措施，体现了邓小平建设有中国特色社会主义理论和党的基本路线的精神，反映了全国各族人民的根本利益和共同愿望，经过努力是能够实现的。会议决定批准《[国民经济和社会发展“九五”计划和2010年远景目标纲要](https://www.pkulaw.com/chl/e2a1d33cf1d17c23bdfb.html)》，批准李鹏总理的报告。
　　会议认为，过去的五年，全国各族人民在中国共产党的领导下，坚定不移地走建设有中国特色社会主义道路，较好地完成了国民经济和社会发展第八个五年计划，国民经济和社会发展取得显著成就，国民经济持续快速增长，提前五年实现了到本世纪末国民生产总值翻两番的目标，经济体制改革取得较大进展，对外开放的总体格局基本形成，城乡人民生活继续改善，各项社会事业全面发展。同时，应当清醒地看到，在经济和社会发展中还面临着不少问题和困难，必须高度重视，采取有效措施加以解决。
　　会议认为，今后十五年，是我国改革开放和社会主义现代化建设事业承前启后、继往开来的重要时期。《纲要》是我国经济和社会综合发展的跨世纪宏伟蓝图，是全面完成社会主义现代化建设第二步战略目标并向第三步战略目标迈出重大步伐的行动纲领。全国各族人民、一切国家机关和武装力量、各政党和各社会团体、各企业事业组织，都必须以高度的历史责任感和时代紧迫感，齐心协力，扎实工作，保证《纲要》的顺利实施。
　　会议认为，实现《纲要》提出的奋斗目标，必须始终坚持邓小平建设有中国特色社会主义理论和党的基本路线，坚持“抓住机遇、深化改革、扩大开放、促进发展、保持稳定”的基本方针，认真贯彻《纲要》提出的指导国民经济和社会发展的重要方针，正确处理改革、发展、稳定的关系，积极推进经济体制和经济增长方式的根本转变，充分发挥市场机制的作用，加强和改善宏观调控，切实解决好关系改革和发展全局的重大问题。
　　会议认为，要保持国民经济持续、快速、健康发展，必须把农业放在发展国民经济的首位。要认真贯彻[农业法](https://www.pkulaw.com/chl/935a352c1b129016bdfb.html)，切实加强农业，增加对农业的投入，努力改善农业生产基本条件，保证粮棉油等基本农产品稳定增产，全面发展和繁荣农村经济。要实施科教兴农战略，积极推广行之有效的先进技术，加强农田水利建设，依法保护耕地，搞好粮棉生产基地建设，加强农业综合开发，加大扶贫工作力度。要继续深化农村改革，稳定和完善以家庭联产承包为主的生产责任制和统分结合的双层经营体制，有条件的地方逐步推进农业适度规模经营。要因地制宜地进一步发展乡镇企业，积极引导农村剩余劳动力的有序转移。要高度重视维护农民的合法权益，逐步缩小工农业产品价格剪刀差，减轻农民负担，增加农民收入，调动和保护农民的积极性，巩固工农联盟。
　　会议要求，要积极推进产业结构的调整，继续加强基础设施和基础工业，大力振兴支柱产业，积极发展第三产业。要继续把抑制通货膨胀作为宏观调控的首要任务，保持宏观经济的稳定，实现财政收支基本平衡。要引导区域经济协调发展，促进全国经济布局合理化，在鼓励东部地区继续发挥优势的同时，更加重视和支持中西部地区的发展，积极朝着缩小差距的方向努力。在经济发展和劳动生产率提高的基础上，不断提高城乡人民的生活水平。要加强国防现代化建设，增强国防实力。
　　会议强调，要以企业改革为中心积极推进经济体制改革。要从实际出发，进一步解放思想，勇于实践，积极探索，加快建立现代企业制度。国有企业改革，要着眼于搞好整个国有经济，突出重点，分类指导。要把企业改革、改组、改造和加强管理结合起来，把企业内部改革和外部配套改革结合起来，全心全意依靠工人阶级办好企业。要积极推进集体企业的改革与发展，不断壮大集体经济。继续发展个体、私营等非公有制经济，加强引导、监督和管理，发挥其对社会主义公有制经济的补充作用。要在国家法律的规范下，积极培育和发展统一开放、公平竞争的市场体系。要坚持和完善按劳分配为主体、多种分配方式并存的分配制度，合理调节个人收入分配，加快社会保险制度改革，建立健全适合我国国情的社会保障体系。要按照发展社会主义市场经济的要求，认真转变政府职能，提高管理水平和工作效率，增强国家宏观调控能力。要继续实行对外开放，充分利用国内和国际两种资源、两个市场，进一步扩大对外开放程度，提高对外开放水平。
　　会议要求，要认真实施科教兴国战略和可持续发展战略。要加快科技进步，优先发展教育，调动科教人员的积极性，促进科技、教育与经济紧密结合。各级政府要继续把计划生育工作摆到重要位置，坚持计划生育的基本国策，严格控制人口增长。要切实保护生态环境，合理开发利用资源，使经济建设与资源、环境相协调。
　　会议强调，必须把社会主义精神文明和民主法制建设提到更加突出的地位，促进社会全面进步。要加强思想道德和文化建设，继续大力开展爱国主义、集体主义、社会主义思想教育。要发展卫生体育事业，提高国民身体素质。要继续推进政治体制改革，建设和完善社会主义民主政治，保障人民当家作主的权利。要坚持和实行依法治国，积极推进社会主义法制建设的进程，加强立法，严格执法，不断提高广大干部和群众的法律意识和法制观念，努力建设社会主义法制国家。要加强勤政廉政建设和反腐败斗争。各级国家机关和国家工作人员都要密切联系群众，全心全意为人民服务，勤奋工作，不尚空谈，克服官僚主义、形式主义，制止弄虚作假、虚报浮夸，厉行勤俭节约，反对铺张浪费。要采取有力措施，依法严厉打击各种刑事犯罪活动，搞好社会治安综合治理，维护社会稳定和国家安全。要维护和发展平等、团结、互助的社会主义民族关系，加大对民族地区的扶持力度，坚持和完善民族区域自治制度，维护祖国统一，促进全国各民族的共同繁荣。
　　会议指出，我国将相继对香港和澳门恢复行使主权，这是实现祖国统一大业的重要里程碑。要积极做好各项准备工作，保证香港和澳门政权的平稳过渡和顺利交接，保持香港和澳门的长期繁荣与稳定。要按照“和平统一、一国两制”的方针，积极推进台湾海峡两岸关系的发展，促进祖国统一大业。台湾问题纯属中国内政，外国势力不得以任何借口和形式进行干涉。中国政府和人民有决心、有能力维护国家的主权和领土完整，绝不允许把台湾从祖国分裂出去。会议呼吁，包括台湾同胞在内的全体中国人携起手来，为早日实现祖国的完全统一而共同努力。
　　会议指出，当前世界仍处在复杂而深刻的变化中。世界要和平，国家要稳定，经济要发展，人类要进步，已经成为当今世界的主旋律。我们将继续坚定不移地奉行独立自主的和平外交政策，发展同世界各国的友好合作关系，为建立公正与合理的国际政治经济新秩序而努力。中国是维护世界和平与地区稳定的重要力量。中国人民将与各国人民一道，为建设一个更加美好的世界作出自己的贡献。
　　会议强调，1996年是实施“九五”计划的第一年。努力做好今年的各项工作，对于完成“九五”计划和实现2010年远景目标，具有重要意义。各级政府要紧紧围绕经济体制和经济增长方式的转变，安排和开展各方面的工作，把已经确定的各项任务和方针政策真正落到实处。
　　会议号召：全国各族人民在邓小平建设有中国特色社会主义理论和党的基本路线指引下，紧密团结在以江泽民同志为核心的党中央周围，万众一心，励精图治，艰苦创业，开拓进取，为全面完成《纲要》确定的宏伟任务而努力奋斗！

关于[国民经济和社会发展“九五”计划和2010年远景目标纲要](https://www.pkulaw.com/chl/e2a1d33cf1d17c23bdfb.html)的报告
（--1996年3月5日在第八届全国人民代表大会第四次会议上　国务院总理　李鹏）

各位代表：
　　制定一个跨世纪的宏伟纲领，继续把改革开放和社会主义现代化建设事业推向前进，是全国人民关心的大事。中国共产党十四届五中全会通过的《[中共中央关于制定国民经济和社会发展“九五”计划和2010年远景目标的建议](https://www.pkulaw.com/chl/331672532195088ebdfb.html)》，提出了全面实现第二步战略目标，并向第三步战略目标迈进的指导方针和主要任务。国务院根据《建议》精神，广泛征求各方面意见，制定了《国民经济和社会发展“九五”计划和2010年远景目标纲要（草案）》。现在，我代表国务院向大会报告，请各位代表连同《纲要（草案）》一并审议，并请全国政协委员提出意见。
　　**一、**“八五”时期国民经济和社会发展的回顾
　　过去的五年，是我国人民沿着建设有中国特色社会主义道路阔步前进的五年。以1992年邓小平同志重要谈话和中共十四大为标志，改革开放和现代化建设进入新的发展阶段。在中国共产党领导下，经过各族人民的共同努力，“八五”计划提出的主要任务已经完成或超额完成，国民经济和社会发展取得显著成就，社会生产力、综合国力和人民生活，都上了一个新的台阶。
　　国民经济持续快速增长。“八五”期间，国民生产总值年均增长12%，1995年达到57600多亿元。我在这里向大会报告：原定2000年比1980年翻两番的目标，已经提前五年实现了。五年来，农村经济全面发展，农业年均增长4.1%，乡镇企业保持发展势头。工业年均增长17.8%，产品结构调整加快，煤炭、电力、钢铁、汽车、化纤、化肥、家用电器都有较大增长。石油天然气和有色金属工业取得新的成绩。轻纺产品供应充裕，花色品种增多。重点建设成绩显著，建成投产大中型基建项目840多个，交通、通信和能源建设得到加强。铁路正线铺轨总里程11000多公里，贯穿南北的京九铁路提前两年全线铺通。高等级公路、港口、机场建设发展较快。新增发电装机总量7000多万千瓦。邮电事业迅速发展，电话交换机总容量新增5800多万门。地质勘查取得新的成绩。基础工业和基础设施建设的成就，缓解了经济增长的“瓶颈”制约，为今后经济发展增添了新的力量。
　　经济体制改革取得突破性进展。按照建立社会主义市场经济体制的目标加快了改革步伐。以分税制为核心的新财政体制，以增值税为主体的新税制，已经基本建立并正常运行。政策性金融和商业性金融初步分开，汇率顺利并轨。新的宏观调控体系的框架初步建立，加强和改善宏观调控取得明显成效。价格进一步放开，市场在资源配置中的基础性作用明显增强。国有企业和农村改革，计划、投资、流通、社会保障体制改革，以及住房制度改革和政府机构改革，都取得新的进展。经过十七年的改革，以公有制为主体、多种经济成分共同发展的格局已经形成。国民经济市场化、社会化程度明显提高，经济活力显著增强。改革的推进，为国民经济和社会发展提供了强大动力，也为本世纪末初步建立社会主义市场经济体制奠定了基础。
　　对外开放的总体格局基本形成。“八五”时期，进一步扩大了对外开放的范围和规模，形成了由沿海到内地、由一般加工工业到基础工业和基础设施的总体开放格局。进出口总额累计超过1万亿美元，比“七五”时期增长一倍以上。实际利用外资超过1600亿美元，其中外商直接投资占70%，投资结构有所改善。引进先进技术和管理经验，促进了国内生产技术和管理水平的提高。国际旅游业迅速发展。1995年末国家外汇储备730多亿美元，国际支付能力增强。对外开放的扩大，促进了国内经济发展，推动了经济体制改革，也增进了同各国政府和人民之间的交往。
　　城乡人民生活继续改善。“八五”期间，扣除物价因素，城镇居民人均生活费收入年均增长7.7%，农村居民人均纯收入年均增长4.5%，社会消费品零售总额年均增长10.6%。1995年末居民储蓄存款余额接近3万亿元，比“七五”末增加两万多亿元。城乡劳动就业不断增加。脱贫工作取得很大成绩，贫困人口由“七五”末的8500万减少到6500万。城乡新建住房43亿平方米，人均居住面积扩大。城镇实行了每周五天工作制。城乡人民文化生活进一步丰富，生活质量得到提高，正在向小康目标前进。
　　各项社会事业全面发展。科技和教育事业在改革中继续前进。五年取得国家级科研成果16万项，科技成果向现实生产力的转化加快。普及九年义务教育取得明显成效，中等职业教育有较快发展，高等教育体制改革迈出较大步伐。计划生育成绩显著，人口自然增长率由1990年的14.39‰下降到1995年的10.55‰，人口增长速度过快的势头得到初步控制。文学艺术、广播影视、新闻出版和社会科学都取得新的进步，思想教育和宣传工作得到加强。卫生体育、环境保护和侨务工作，以及妇女、儿童、老龄和残疾人工作都取得新的成绩。加强了勤政廉政建设、反腐败斗争和社会治安综合治理，查处了一批大案要案。社会主义精神文明和民主法制建设取得进展，安定团结的局面进一步巩固。国防建设得到加强，中国人民解放军、武装警察部队和公安干警在保卫祖国、维护国家安全和社会稳定中作出了新的贡献。
　　“八五”时期取得巨大成就的根本原因，在于坚定不移地走建设有中国特色的社会主义道路，坚持党的基本路线不动摇。在国际风云剧变的形势下，全国人民团结奋斗，紧紧抓住经济建设这个中心不放，坚持四项基本原则，坚持改革开放，实现了经济发展、政治稳定、民族团结、社会进步。在以江泽民同志为核心的党中央领导下，我们伟大的社会主义祖国，巍然屹立在世界的东方！
　　各位代表！在肯定成绩的同时，我们也看到，前进中还有不少问题和困难。突出的是，出现了比较严重的通货膨胀，五年零售物价年均上涨11.4%。国有企业生产经营困难较多，管理制和经营机制不适应社会主义市场经济的要求。农业仍然是国民经济的薄弱环节，不能满足经济发展和人民生活提高的需要。收入分配关系还没有理顺，部分社会成员收入悬殊。经济秩序还比较混乱。有些腐败现象仍在蔓延滋长。一些地方社会治安状况不好。社会主义精神文明和民主法制建设也面临不少新的问题。这些问题的产生，虽然有客观原因，但也反映了政府工作中的缺点和不足。我们要认真总结经验，改进工作，在改革和发展中采取有效措施，切实加以解决。

　　**二、**今后十五年的奋斗目标和指导方针
　　今后十五年，是我国改革开放和社会主义现代化建设事业承前启后、继往开来的重要时期。经过建国以来的建设，特别是改革开放以来的快速发展，我国已经有了比较雄厚的物质技术基础。广阔的国内市场和较高的储蓄率，蕴藏着巨大的发展潜力。深化改革和扩大开放，将为经济发展注入新的活力。社会稳定和民族团结，为现代化建设提供了基本保证。更重要的是，在实践中形成和发展起来的建设有中国特色社会主义的理论、路线和方针，为继续前进指明了方向。从世界范围看，和平的国际环境和良好的周边关系可望继续保持，我国仍有可能集中力量进行经济建设。世界科技进步和产业结构的调整，亚太地区经济的迅速增长，给我国经济发展提供了有利的条件。在我国中长期发展中，也有不少制约因素。突出的是：人口和就业负担较重，人均资源相对不足，国民经济整体素质低；在日趋激烈的国际竞争中，面临着发达国家在经济与科技上占优势的压力，在国际关系中面临着霸权主义和强权政治的压力。综观世纪之交的国内外形势，我们面前有不可多得的历史机遇，也存在严峻的挑战。我们必须居安思危，奋发图强，在现代化道路上迈出更大步伐！
　　未来十五年的主要奋斗目标是：“九五”时期，全面完成现代化建设的第二步战略部署，2000年在人口将比1980年增长3亿左右的情况下，实现人均国民生产总值比1980年翻两番；基本消除贫困现象，人民生活达到小康水平；加快现代企业制度建设，初步建立社会主义市场经济体制。2010年，实现国民生产总值比2000年翻一番，使人民的小康生活更加宽裕，形成比较完善的社会主义市场经济体制。在推进改革和发展的同时，社会主义精神文明和民主法制建设要取得显著进展，实现社会全面进步。这个奋斗目标，展现了本世纪末、下世纪初我国现代化建设的美好前景，表达了中华民族自立自强的雄心壮志。实现了这个目标，我国社会生产力、综合国力和人民生活水平都将再上一个大台阶，社会经济面貌将发生历史性的巨大变化，为下世纪中叶基本实现现代化，奠定坚实的基础。这个目标是宏伟的，经过努力也是一定能够实现的！
　　实现上述目标，必须始终坚持邓小平建设有中国特色社会主义的理论和党的基本路线，解放思想，实事求是，遵循“抓住机遇、深化改革、扩大开放、促进发展、保持稳定”的基本方针，认真贯彻中共中央《建议》提出的指导国民经济和社会发展的九条重要方针。这九条重要方针是：保持国民经济持续、快速、健康发展；积极推进经济增长方式转变，把提高经济效益作为经济工作的中心；实施科教兴国战略，促进科技、教育与经济紧密结合；把加强农业放在发展国民经济的首位；把国有企业改革作为经济体制改革的中心环节；坚定不移地实行对外开放；实现市场机制和宏观调控的有机结合，把各方面的积极性引导好、保护好、发挥好；坚持区域经济协调发展，逐步缩小地区发展差距；坚持物质文明和精神文明共同进步，经济和社会协调发展。
　　根据中共中央《建议》提出的一系列指导方针，在制定《纲要（草案）》过程中，着重考虑了以下几点：
　　第一，正确处理改革、发展、稳定的关系。发展是硬道理，中国解决所有问题的关键要靠自己的发展。改革是发展的动力，只有继续深化改革，才能解决妨碍经济社会发展的深层次矛盾。保持政治和社会稳定是推进改革和发展的基本前提，稳定又必须通过深化改革和不断发展来实现。今后十五年，改革、发展、稳定都面临着新的形势和任务，处理好三者关系至关重要。发展要注重提高质量、效益和优化结构，改革要在重点和难点问题上取得突破性进展，这都会引起利益格局和社会关系的变化，必须高度重视保持政治和社会的稳定。要善于从整体上把握三者之间的关系，使之相互协调，相互促进。
　　第二，积极推进经济体制和经济增长方式的根本转变。从计划经济体制向社会主义市场经济体制转变，经济增长方式从粗放型向集约型转变，这是实现今后十五年奋斗目标的关键所在。经济体制转变要遵循市场经济的一般规律，同时坚持社会主义方向。经济增长方式转变，要处理好速度和效益的关系，提高经济整体素质和生产要素的配置效率，注重结构优化效益、规模经济效益和科技进步效益。我国经济规模已经很大，每年新增加的投入相当可观，但在生产、建设和流通等领域，经济效益低的问题十分突出。必须通过深化改革，形成有利于节约资源、降低消耗、提高质量、增加效益的企业经营机制，有利于自主创新的技术进步机制，有利于市场公平竞争和资源优化配置的经济运行机制。转变经济增长方式归根到底要靠加快科技进步，提高劳动者素质。“九五”时期，要充分利用现有基础，能够以现有企业为依托，通过改革、改组、改造或扩建提高生产能力的，就不要再铺新摊子。新建项目要提高技术起点，注意经济规模和效益。各行各业都要通过加强科学管理，注重资源节约和综合利用，减少资源的占用和消耗。认真实行两个转变，把经济增长潜力充分发挥出来，我国经济和社会发展就能够取得更好的成绩。
　　第三，认真解决关系改革和发展全局的重大问题。主要是：加强农业基础问题，搞好国有企业改革和发展问题，抑制通货膨胀、保持宏观经济稳定问题，发展教育和科技问题，控制人口、保护资源和环境问题，缩小地区发展差距和理顺分配关系问题，加强社会主义精神文明和民主法制建设问题，勤政廉政建设、反腐败斗争问题，以及社会治安综合治理问题。江泽民主席在《正确处理社会主义现代化建设中的若干重大关系》的重要讲话中，提出了解决这些问题的基本思路和指导思想。要根据中共中央《建议》和江泽民主席讲话的精神，采取有力的政策措施，努力把这些问题解决好。
　　第四，计划要体现发展社会主义市场经济的要求。《纲要（草案）》是我国在发展社会主义市场经济条件下的第一个中长期计划，要注重市场在国家宏观调控下对资源配置的基础性作用，突出国家计划的宏观性、战略性和政策性，发挥社会主义能够集中力量办大事的优势。计划指标总体上是预测性、指导性的，着重提出经济、社会发展的方向、任务以及相应的发展战略和措施，提出反映经济社会发展和结构变化的总量指标，以及若干具有全局意义的重大项目，其他一些指标和项目在今后的年度计划中安排。《纲要（草案）》重点放在“九五”，后十年只提出大体的轮廓，使之与下世纪初叶的发展相衔接，以保持三步走战略的连续性。

　　**三、**促进国民经济持续、快速、健康发展
　　关于“九五”和后十年经济建设的主要任务和战略布局，我着重讲以下几个问题：
　　第一，确保农业和农村经济持续稳定增长。这是今后十五年经济发展中非常重要而又难度最大的一项任务。为了满足经济发展和人民生活的需要，一定要保证粮、棉、油等基本农产品稳定增产。我国以占世界7%的耕地，养育着世界22%的人口，粮食生产有特殊的重要性。2000年粮食总产量要保证达到4900亿公斤，力争达到5000亿公斤。我国粮食增产潜力很大，能够立足国内解决自己的吃饭问题。采取的政策措施主要是：实施科教兴农战略，重视农村科技队伍建设，推广一系列行之有效的先进适用技术。加强大江大河大湖治理，疏浚中小河流，增强抗御水旱灾害的能力。大力改造中低产田，搞好黑龙江、吉林、新疆、黄淮海等地区的粮棉生产基地建设，继续扶持粮棉集中产地发展经济。加快发展农用工业，增加农业生产资料的供应，提高农业机械化和现代化水平。坚持和完善“米袋子”省长负责制、“菜篮子”市长负责制。要依法保护耕地，建立健全基本农田保护制度，珍惜和合理利用每一寸土地。积极发展节水型农业和节粮型畜禽养殖。鼓励农村种植业、养殖业、加工业的有机结合，推动农工贸一体化，促进农业向高产、优质、高效方向发展。搞好农业综合开发，全面发展林、牧、副、渔各业。从中央到地方，各级政府都要增加对农业的投入，鼓励和引导农村集体、农民个人和社会各方面增加投入。要充分利用农村人力资源，开展农田基本建设，兴修水利，修筑道路，植树造林，改善农业和农村经济发展的条件。
　　乡镇企业是中国农民的伟大创造。乡镇企业的改革和发展，是繁荣农村经济、扩大就业和增加农民收入的重要途径。要充分利用当地资源优势，积极发展农产品加工和为农服务的产业。注意节约资源，防治和减少环境污染。为了创造良好的投资环境和提高经济效益，发展乡镇企业宜相对集中，并与小城镇建设结合起来。要积极引导农业剩余劳动力有序转移。
　　继续深化农村改革，稳定和完善以家庭联产承包为主的生产责任制和统分结合的双层经营体制，有条件的地方要逐步实行农业适度规模经营。继续推进国有农场的改革和发展。进一步理顺农产品和农业生产资料价格，建立健全以批发市场为中心的农产品市场体系。积极发展农村社会化服务，办好供销合作社和信用合作社等合作经济组织，不断壮大集体经济。必须高度重视维护农民合法权益，严格执行现行的规定，采取切实措施减轻农民负担，稳定增加农民收入，调动和保护广大农民的积极性，巩固工农联盟。
　　第二，积极推进产业结构的调整。要继续加强基础设施和基础工业，大力振兴支柱产业，积极发展第三产业。
　　基础设施和基础工业建设要与国民经济发展相适应。今后十五年，国家要集中必要的力量，在水利、能源、交通、通信和重要原材料工业方面，建设一批大型工程，包括：长江三峡和黄河小浪底水利枢纽工程，南水北调工程，山西、陕西、内蒙古煤炭基地，南昆铁路、南疆铁路和神黄铁路，公路国道主干线，通信光缆干线网络，以及一批大型港口、机场等。这些重要工程，有的是“九五”建设的，有的是“九五”做准备，下世纪初开始建设的。基础设施和基础工业投资多，建设周期长，要统筹规划，合理布局，突出重点，避免盲目发展和重复建设。
　　要根据市场需求，振兴机械、电子、石油化工、汽车和建筑等支柱产业，以带动整个经济的增长。发展支柱产业，要提高技术起点，在引进先进技术的同时，增强自主开发和创新能力，形成经济规模，注重经济效益。
　　发展轻纺工业，对满足人民生活需要，扩大出口，积累建设资金，具有重要意义。要适应国内外市场变化，加快产品结构调整，提高产品质量，增加花色品种，增强竞争能力。
　　积极发展第三产业，要以第一、第二产业的发展为基础，形成合理的布局和结构。继续发展商业和生活服务业，发展旅游业以及信息、咨询等中介服务产业，规范和发展金融、保险业，引导房地产业健康发展。进一步发挥第三产业在提高社会生产效率、增加城乡劳动就业和方便人民生活等方面的重要作用。
　　第三，促进区域经济协调发展。总的要求是，按照统筹规划、因地制宜、发挥优势、分工合作、协调发展的原则，正确处理全国经济总体发展与地区经济发展的关系，正确处理发展区域经济与发挥各省（自治区、直辖市）积极性的关系。要按照市场经济规律，以中心城市和交通要道为依托，进一步形成和发展若干突破行政区划界限的经济区域。
　　地区发展不平衡是我国的一个基本国情，也是大国经济发展进程中的普遍现象。改革开放以来，全国各地经济都有很大发展，但由于发展快慢不同，地区差距有所扩大。在“九五”期间，要更加重视支持中西部地区的发展，积极朝着缩小差距的方向努力。国家采取的政策措施包括：加强中西部地区资源勘查，优先安排资源开发和基础设施建设项目，逐步增加财政支持和建设投资；调整加工业的布局，引导资源加工型和劳动密集型的产业向中西部地区转移；理顺资源性产品的价格，增强中西部地区自我发展的能力；改善中西部地区的投资环境，引导外资更多地投向中西部地区；加强东部地区与中西部地区的经济联合与合作，鼓励向中西部地区投资，引导人才向中西部流动。东北等老工业基地要加快改造和调整的步伐，充分发挥它的作用，国家给予必要的支持。地区差距是历史形成的，缩小差距需要有个过程。从根本上说，有条件的地区发展得快一些，有利于增强国家经济实力，支持欠发达地区发展经济。东部地区要发挥已有的优势，在转变经济增长方式、推动产业结构升级、发展外向型经济和促进经济健康发展方面，为全国提供新的经验。中西部地区的发展潜力很大，只要加快改革开放步伐，增强经济活力，就一定能够加快发展，有些地区甚至会后来居上。
　　第四，努力保持宏观经济的稳定。现在物价上涨幅度仍然偏高，加之“九五”时期要继续理顺价格关系，物价上涨的压力很大，必须把抑制通货膨胀作为宏观调控的首要任务，避免经济出现大的波动。根据对各方面条件的综合分析，“九五”期间的宏观调控目标，年均经济增长速度为8%左右，固定资产投资率为30%，物价上涨幅度明显降低，首先要努力使之低于经济增长率。要继续加强和改善宏观经济调控。保持合理的固定资产投资规模和在建规模，加大投资结构调整力度，提高投资效益。继续实行适度从紧的财政政策和货币政策。现在国家财政困难，作为振兴财政的第一步，要继续完善税制，调整有关税率，扩大税源基础，取消税收减免，加强税收征管，努力增收节支，逐步减少财政赤字，实现财政收支基本平衡。要适当控制货币供应总量，保持币值的稳定。根据产业政策和信贷原则调整贷款结构，提高资金使用效率。保持国际收支基本平衡，进一步增强国际支付能力。
　　第五，不断提高城乡人民生活水平。这是我国现代化建设的根本出发点和归宿。“九五”时期，扣除物价上涨因素，预计城镇居民人均生活费收入年均增长5%左右，农民人均纯收入年均增长4%左右。要积极拓宽城乡就业渠道，不断扩大就业和再就业。注重提高生活质量，把解决居民住房问题放在突出的位置，同时努力改善交通、通信、供水条件和生活环境，发展社会服务，加强公共福利设施建设，丰富城乡人民文化生活。这些年来，我国经济虽有很大发展，但至今还是一个发展中国家，靠全体人民再经过几十年努力，才能基本实现现代化。要合理引导消费，形成适合我国国情的消费结构和消费方式，把生活改善建立在经济发展和劳动生产率提高的基础上。
　　解决贫困人口的生活困难特别是农村贫困人口的温饱问题，是“九五”时期的一项艰巨任务。贫困地区要发扬艰苦创业精神，主要依靠自己的力量脱贫致富。从中央到地方，都要加大扶贫工作的力度，认真落实扶贫攻坚计划，继续增加并管好用好各项扶贫资金，加强开发性扶贫。动员全社会关心扶贫工作，以多种形式支持和促进贫困地区经济社会发展。帮助遭受严重自然灾害地区的群众生产自救和重建家园。重视扶持革命老区的经济发展。逐步建立城市居民最低生活保障制度，帮助城市贫困人口解决生活困难。
　　各位代表！国防现代化是我国现代化建设的一项重要任务。为了保卫国家安全，贯彻积极防御的战略方针，走有中国特色的精兵之路，必须加强国防现代化建设，增强国防实力。要重视科技强军，加强国防科学技术研究，把武器装备的发展放在依靠自己力量的基础上，优先发展高技术条件下防卫作战所需要的武器装备，着重加强新型武器装备的研制。继续调整国防科研和国防工业结构，实行军民结合、平战结合，运用军工高技术发展船舶、飞机、卫星等民用产品，逐步建立适应社会主义市场经济发展的国防工业运行机制和国防动员体系。加强人民武装警察部队建设。开展国防教育，增强国防观念。加强拥政爱民、拥军优属工作，巩固军政、军民团结。

　　**四、**以企业改革为中心积极推进经济体制改革
　　本世纪末初步建立社会主义市场经济体制，有许多问题需要解决，任务艰巨，时间紧迫。要以更大的决心和魄力，积极探索，勇于创新，扎扎实实地推进改革和开放。
　　第一，建立现代企业制度，搞好国有企业的改革和发展。这是初步建立社会主义市场经济体制的关键。要按照“产权清晰、权责明确、政企分开、管理科学”的要求，在“九五”末使大多数国有大中型骨干企业建立起现代企业制度。继续抓好中央和地方确定的企业试点工作，务必在重点、难点问题上取得突破。在解决企业内在机制、外部环境和历史遗留问题方面统筹考虑，配套推进。对实践中的成功经验，要及时总结推广，促进面上企业的改革。城市改革试点要与企业改革结合起来。
　　国有企业改革，要着眼于搞好整个国有经济，突出重点，分类指导。国家近期要集中力量抓好1000户国有大型企业和企业集团的改革与发展，使之成为自主经营、自负盈亏、自我发展、自我约束的法人实体和市场竞争主体，发挥它们在国民经济中的骨干作用。省、自治区、直辖市也要抓好一批国有骨干企业。“九五”期间国家将安排一笔资金，用于鼓励企业兼并，冲销破产企业债务，把相当一部分“拨改贷”形成的债务转为国家资本金，降低企业资产负债率。所有企业都要面向市场，建立商业信誉，减少和避免资金拖欠。放活国有小企业，可以区别不同情况，采取改组、联合、兼并、股份合作制、租赁、承包经营和出售等形式。从一些地方的实践看，国有小企业经过改革改组，绝大部分仍然是国有经济或者集体经济，即不同形式的公有经济，出售给私营企业或个人的是少数。
　　要把企业改革、改组、改造和加强管理结合起来，把企业内部改革和外部配套改革结合起来。所有企业，都要全心全意依靠工人阶级，建设好领导班子，转变经营机制，建立科学的管理制度，充分挖掘内部潜力，加快技术进步，生产适销产品，提高经济效益。对企业领导者，既要保证其对生产经营的指挥权，又要建立健全必要的监督制度。要制定和落实深化企业改革的综合配套措施，积极创造条件，多渠道分流企业富余职工，分离企业承担的社会服务职能。城乡集体经济是公有制经济的重要组成部分，要积极推进集体企业的改革与发展。继续发展个体、私营等非公有制经济，加强引导和管理，发挥其有益的补充作用。
　　第二，积极培育统一开放、竞争有序的市场体系。发展社会主义市场经济，必须采取积极而义稳妥的步骤，形成比较完善的金融市场和房地产、劳动力、技术、信息等要素市场。要继续完善商品市场，发展连锁经营和代理制等新的营销方式。改进粮食等重要产品的购销和储备制度。加强市场管理和质量监督，整顿流通秩序，创造公平竞争环境，保护生产者和消费者的合法权益。
　　要进一步改革投资体制，确定投资主体，建立资本金制度，逐步发展市场融资方式。由企业作为投资主体的，企业和金融机构承担投资的风险和责任。由政府机构或社会公益机构作为投资主体的，也要明确风险和责任。新开工建设的生产经营项目都要实行项目法人责任制。国家确定的重点建设新项目要逐步向社会招标。
　　第三，调节个人收入分配，建立健全社会保障体系。坚持和完善按劳分配为主体、多种分配方式并存的制度，按照效率优先、兼顾公平的原则，建立合理的工资形成机制，逐步完善适合企事业单位和行政机关的工资制度。国家保护合法收入，取缔非法收入，打击不正当竞争，通过完善个人所得税，以及开征其他必要税种等措施，调节过高收入。运用法律手段和分配政策，协调城乡之间、地区之间、行业之间、不同社会群体之间的分配关系，逐步解决社会分配差距过大的问题。
　　“九五”期间，要加快养老保险、失业保险和医疗保险制度改革，发展社会救济、社会福利、优抚安置、社会互助、个人积累等多层次的社会保障，初步形成适合我国国情的社会保障制度。城镇职工养老和医疗保险金由国家、单位和个人共同负担，实行社会统筹和个人帐户相结合。积极发展商业保险，发挥其对社会保障的补充作用。扩大失业保险的覆盖面，建立失业救济和再就业相结合的制度。多渠道筹措并切实管好用好各类社会保障资金。推进住房制度改革，建设“安居工程”，加快住房商品化的步伐。
　　第四，转变政府职能，增强国家宏观调控能力。各级政府都要按照发展社会主义市场经济的要求，认真转变职能，实行政企职责分开，加强政府部门自身建设，精简机构，提高管理水平和工作效率。中央政府主要运用经济手段和法律手段，并辅之以必要的行政手段，对国民经济实行宏观调控。要深化计划体制改革，通过制定与实施发展战略、宏观调控目标和经济政策，保持经济总量的基本平衡，促进经济结构优化。要健全财政职能，提高财政收入占国民生产总值的比重，以及中央财政收入占全国财政收入的比重，逐步实行规范的转移支付制度，统一管理政府的国内外债务。严肃财经纪律，加强对预算外资金的管理。强化中央银行对货币供应量的调控职能，加强对金融机构的监管，防范金融风险。进一步调整和改革政府机构，把综合经济部门逐步调整和建设成为职能统一、具有权威的宏观调控部门；把专业经济管理部门逐步改组为不具有政府职能的经济实体，或改为国家授权经营国有资产的单位，或改为行业管理组织；其他政府部门也要进行合理调整。要建立权责明确的国有资产管理、监督和营运体制，促进国有资产保值增值。
　　第五，提高对外开放水平。要充分利用国内和国际两种资源、两个市场，进一步扩大对外开放。“九五”期间，要适应社会主义市场经济发展的需要，按照国际经济通行规则，初步建立统一规范的对外经济体制：国家对经济特区和上海浦东新区的基本政策不变，在发展社会主义市场经济的过程中，有些具体办法要有所调整和完善。经济特区要增创新优势，更上一层楼；经济特区、沿海开放城市和开放地带要积极参与国际经济合作，充分发挥示范，辐射和带动作用。沿交通干线、沿江、沿边地区和内陆中心城市要发挥各自优势，切实推进对外开放，促进经济开发和振兴。要根据改革和发展的要求，逐步开放国内市场。有步骤地开放金融、商业、旅游等服务领域。搞好智力引进工作。
　　坚持市场多元化战略，调整和改善进出口贸易结构，着重提高出口商品的质量和附加值，加强售后服务。巩固现有市场，开拓新的市场，拓宽出口渠道。坚持统一政策、平等竞争、工贸结合、推行代理制的方向，继续改革和完善外贸管理体制。
　　继续积极、合理、有效地利用外资，把重点放到提高成效和水平上来。逐步对外资企业实行国民待遇，规范税制，公平税负，为中外企业创造平等竞争条件。积极引导外资参与能源、交通、农业等基础性项目建设和老企业技术改造。
　　实行对外开放是我国坚定不移的方针。发展对外经济技术合作不仅对中国有利，也是中国对世界经济发展的积极贡献。某些西方国家处心积虑地把中国排斥在世界贸易组织之外，违背这个世界性组织的宗旨，也有损于它的普遍性和公正性。人们将会看见，中国同世界各个国家和地区经济贸易关系的发展，终究是任何力量也阻挡不了的。

　　**五、**实施科教兴国战略和可持续发展战略
　　实施这两大战略，对于今后十五年的发展乃至整个现代化的实现，具有重要意义。要加快科技进步，优先发展教育，控制人口增长，合理开发利用资源，保护生态环境，实现经济社会相互协调和可持续发展。
　　面向经济建设，加快科技进步。在当代条件下，科学技术作为第一生产力的作用越来越突出，成为推动经济社会发展和国家强盛的决定性因素。经济建设必须依靠科学技术，科学技术工作必须面向经济建设。一是促进技术开发和应用，加速科技成果商品化和产业化，自主开发和引进、消化先进技术相结合，集中力量解决经济社会发展中的重大和关键技术问题。二是积极发展高新技术及其产业，在一些重要领域接近或达到国际先进水平，应用高新技术改造传统产业，切实办好高新技术产业开发区。三是加强基础性科学研究，瞄准世界科技前沿，攀登科技高峰，力争在优势领域有所突破。在社会发展领域，要加强计划生育、重大疾病防治、环境保护、资源综合利用和再利用、防灾减灾等技术的研究和应用。深化科技体制改革，促进科研、开发、生产与市场的结合。按照“稳住一头，放开一片”的方针，合理分流人才，优化科研组织结构。加强科研院所、高等院校和企业之间的联合。鼓励大型企业建立技术开发中心，努力使企业成为技术开发的主体。加强知识产权保护，发挥专利制度的作用。
　　优先发展教育，提高国民素质。这是我国现代化事业的百年大计。2000年全国要基本普及九年义务教育，基本扫除青壮年文盲，在经费和师资等方面要予以保证，并加强对贫困地区的支持。高等教育发展规模要适度，着重提高教育质量和办学效益，重点建设好一批高等学校和学科。积极发展多形式、多层次的职业教育和成人教育。优化教育结构，使普通教育和职业教育的比例更加合理。加快教育体制改革，积极探索与我国改革和发展相适应的办学机制和办学模式。鼓励社会力量办学，逐步形成政府办学为主与社会参与办学的新体制。提倡多种形式的联合办学，优化配置教育资源。各级各类学校都要加强和改进思想政治教育，全面提高学生素质。重视师资队伍建设，全面提高教师素质，改善教师的工作、学习条件和住房等生活条件。教育要面向现代化，面向世界，面向未来，培养大批优秀人才。
　　坚持计划生育基本国策，严格控制人口增长。全国人口2000年要控制在13亿以内，2010年要控制在14亿以内，任务艰巨。各级政府要继续把计划生育工作摆到重要位置，坚持和完善目标管理责任制。稳定和认真执行现行的计划生育政策。提倡晚婚晚育，提倡一对夫妇生育一个孩子，做好优生优育工作。深入开展宣传教育，增强全民人口意识。要把计划生育工作的重点放在农村和流动人口，同发展农村经济、脱贫致富和建立文明幸福家庭结合起来。研究人口老龄化问题，并采取相应对策。
　　加强环境、生态保护，合理开发利用资源。这是功在当代、泽及子孙的大事。我国人均耕地、水、森林和不少矿产资源都低于世界人均水平，又处在迅速推进工业化的发展阶段，加上粗放的生产经营方式，资源浪费和环境污染相当严重。随着人口增加和经济发展，这个问题可能更加突出。要依法大力保护并合理开发利用土地、水、森林、草原、矿产和生物等自然资源，千方百计减少浪费。积极开发海洋资源。尽快完善自然资源有偿使用制度和价格体系，建立资源更新的经济补偿机制。坚持经济建设、城乡建设与环境建设同步规划、同步实施、同步发展，所有建设项目都要符合环境保护的要求。各级政府都要依法严格管理环境，特别要加强对工业污染的控制和治理，以及城市环境的整治。积极发展生态农业，加快水土流失地区的综合治理，加强草原建设和防沙治沙，控制农田污染和水污染，努力改善生态环境。到2000年，力争使环境污染和生态破坏加剧的趋势得到基本控制，部分城市和地区的环境质量有所改善。

　　**六、**加强精神文明和民主法制建设
　　我国现代化事业的宏伟目标，是建设富强、民主、文明的社会主义国家。在发展经济的同时，必须把精神文明和民主法制建设提到更加突出的地位，促进社会全面进步。
　　切实加强思想道德和文化建设。在发展市场经济的过程中，在世界各种思想文化相互激荡的条件下，加强社会主义精神文明建设具有特殊重要的意义。精神文明建设包括教育科学文化建设和思想道德建设。各级政府要把精神文明建设纳入社会发展的总体规划，增加投入，给予物质保障，真正做到“两手抓，两手都要硬”。任何时候都不能以牺牲精神文明为代价，换取经济的一时发展。
　　思想道德建设规定着精神文明的性质和方向。要坚持不懈地用邓小平建设有中国特色社会主义的理论教育干部和人民，在全社会形成共同理想和精神支柱，增强民族凝聚力。继续大力开展爱国主义、集体主义、社会主义思想教育，以科学的理论武装人，以正确的舆论引导人，以高尚的精神塑造人，以优秀的作品鼓舞人，培养有理想、有道德、有文化、有纪律的社会主义公民。弘扬中华民族的传统美德，吸取人类文明的积极成果，抵御资本主义和封建主义腐朽思想的侵蚀。重视社会公德、职业道德和家庭伦理道德教育，发展平等、团结、友爱、互助的人际关系。发扬艰苦创业、勤俭节约的优良传统，反对铺张浪费的奢靡之风，提倡科学、文明、健康的生活方式，形成良好的社会风尚。
　　改革开放为文化建设创造了良好的条件，提出了更高的要求。要坚持“为人民服务，为社会主义服务”的方向，坚持“百花齐放，百家争鸣”的方针，弘扬主旋律，提倡多样化，促进文化事业全面发展。文艺工作者要深入生活，努力创作无愧于伟大时代的优秀作品。新闻宣传要坚持正确的舆论导向，激发人们投身现代化事业的积极性。要完善文化经济政策，加强文化市场管理。搞好图书馆、文化馆、档案馆、科技馆和博物馆等文化设施建设，重视保护文物。大力普及科学文化知识，破除迷信，移风易俗。发展社区文化、企业文化、村镇文化和校园文化，丰富基层文化生活。社会科学工作者要以马克思列宁主义、毛泽东思想、邓小平建设有中国特色社会主义的理论为指导，着重研究我国改革、发展中的重大理论和实践问题，为现代化事业作出新贡献。
　　发展卫生体育事业，提高国民身体素质。要实行预防为主的方针，保健和医疗并重。积极发展卫生保健事业，“九五”期间基本做到人人享有初级卫生保健。重视农村医疗卫生工作，发展合作医疗，完善县、乡、村三级医疗保健网，改善农村饮水质量和卫生状况。重视卫生健康教育，加强对传染病、职业病、地方病的防治。振兴中医药事业。积极开发新药，加强医药市场管理。发展体育运动，实施全民健身计划，增强人民体质，提高竞技水平。
　　加强社会主义民主和法制建设。要继续推进政治体制改革，建设和完善社会主义民主政治，保障人民当家作主的权利。各级政府都要自觉接受同级人民代表大会及其常委会的监督，倾听人民群众的呼声。积极支持人民政协的工作，听取民主党派、无党派人士和专家学者的意见。支持工会、共青团、妇联等人民团体的工作，使广大群众的意愿在政府工作中得到充分体现。努力做好侨务工作。建立健全必要的制度和程序，促进决策的科学化、民主化。继续完善城镇居民自治制度和农村村民自治制度，加强基层政权建设，健全基层民主和经济监督，充分调动人民群众的积极性。
　　加强法制建设，依法治国，建设社会主义法制国家，是实现国家长治久安的重要保证。“九五”时期，国务院将根据改革和发展的需要，抓紧起草并提请全国人大常委会审议一批重要法律草案，制定一批行政法规，规范和保障社会、经济健康发展。要加强经济司法和行政执法，严厉打击骗税逃税、走私贩私、金融诈骗、制售假冒伪劣商品、侵犯知识产权等违法犯罪行为，整顿经济秩序。继续实施普法规划，教育全体公民学法守法，增强法制观念和公民意识。各级政府都要依法管理经济和社会事务，坚决维护国家法律法规和政策的统一性与严肃性，做到有法必依，执法必严，违法必究。“九五”期间要完成全国省、县两级勘界工作。
　　加强勤政廉政建设和反腐败斗争。这是关系我国现代化事业成败的一项长期而艰巨的任务。政府机关首先是国务院各部门，领导干部首先是高级干部，要以身作则，廉洁自律，自觉接受监督。国有企事业单位的领导人员，也必须奉公守法。严厉查处违法违纪案件特别是大案要案，坚决纠正部门和行业不正之风。对那些以权谋私、贪赃枉法、行贿受贿的腐败分子，不管职位高低，都要绳之以法，决不姑息养奸。加强司法、监察和审计工作，建立健全勤政廉政制度和监督制约机制，深入开展思想教育，标本兼治，防范和消除腐败。国家公务员要密切联系群众，克服官僚主义、形式主义、弄虚作假和浮夸作风，提高自身素质，勤奋工作，不尚空谈，全心全意为人民服务，以优良的政风推动社会风气的好转。
　　维护社会稳定和国家安全。保持良好的社会治安环境，保障人民群众安居乐业，是各级政府的重要责任。要采取有力措施，严厉打击暴力犯罪、毒品犯罪、流氓恶势力和带黑社会性质的犯罪团伙，以及其他刑事犯罪活动。继续开展“扫黄打非”活动，扫除卖淫、嫖娼等各种社会丑恶现象，净化社会环境。加强社会治安综合治理，坚持领导责任制，专门工作与群众路线相结合，把各项措施落实到基层，把问题解决在萌芽状态。加强流动人口管理，及时解决民事纠纷，正确处理人民内部矛盾，化解社会不稳定因素。加强政法队伍建设，提高干警政治和业务素质。严格执行安全管理制度，防止重大火灾等恶性事故的发生。要提高警惕，防范和及时粉碎一切敌对势力的渗透、颠覆、分裂等各类破坏活动，维护国家安全。
　　各位代表！巩固和发展民族团结，是全国人民的根本利益所在。我国是统一的多民族国家，要维护和发展平等、团结、互助的民族关系，实现各民族共同繁荣和进步。改革开放以来，民族地区社会经济发展加快。“九五”时期，要继续从资金、技术、人才、教育等方面加大扶持力度，促进民族地区改革开放和社会经济发展。经济发展和人民生活的改善，将为进一步巩固民族团结奠定更加牢固的基础。要坚持和完善民族区域自治制度，充分保障自治地方的自治权利和民族平等权利。大力培养民族干部和各类人才。要始终不渝地维护祖国统一和民族团结，坚决反对一切分裂祖国和破坏民族团结的行为。全面贯彻宗教政策，保障公民宗教信仰自由，教育信教群众爱国爱教。依法加强对宗教事务的管理，积极引导宗教与社会主义社会相适应。我国经济和社会发展欣欣向荣的形势，为各民族共同繁荣提供了前所未有的机遇，各族人民团结奋斗，一定能够创造更加美好的未来。

　　**七、**积极促进祖国和平统一大业
　　各位代表！1997年7月1日和1999年12月20日，我国将相继对香港和澳门恢复行使主权。这是实现祖国统一大业的重要里程碑。我国政府将一如既往，贯彻执行“一国两制”的方针，按照[香港特别行政区基本法](https://www.pkulaw.com/chl/5625aa4f893e0672bdfb.html)和[澳门特别行政区基本法](https://www.pkulaw.com/chl/fe7f7ceef711d630bdfb.html)，积极做好各项准备工作，并愿意在中英联合声明和中葡联合声明的基础上，加强同英、葡两国政府的合作，为香港和澳门政权的顺利交接，保持港澳的长期繁荣与稳定而努力。我国在香港和澳门恢复行使主权后，其现行社会经济制度不变，享有高度的自治，继续保持自由港地位，保持香港的国际金融、贸易、航运中心地位。今天是3月5日，距我国对香港恢复行使主权还有483天，海内外同胞正以欢欣鼓舞的心情，期待着这个伟大日子的到来。全国人民代表大会香港特别行政区筹备委员会已经成立，筹组香港特别行政区的工作进入具体落实阶段。这是我国对香港恢复行使主权进程中的一件大事。我们相信，筹委会一定能够广泛听取香港各界人士的意见，在实现香港政权平稳过渡中，胜利完成光荣而神圣的历史使命。
　　多年来，经过台湾海峡两岸同胞的共同努力，两岸人员往来和经济、文化等方面的交流有很大发展。按照“和平统一、一国两制”的方针，结束两岸分离局面，完成统一大业，这是我们的一贯立场。江泽民主席关于促进祖国统一的八项主张，受到海内外的普遍欢迎。我们针对台湾当局某些领导人制造“两个中国”、“一中一台”和“台湾独立”的图谋所进行的斗争，显示了维护国家主权和领土完整的决心和能力。台湾问题纯属中国内政，外国势力不得以任何借口和形式进行干涉。我们主张并且一贯致力于和平统一，但不承诺放弃使用武力。这不是针对台湾同胞的，而是针对外国势力干涉中国统一和搞“台湾独立”图谋的。台湾作为中国一部分的地位绝不容许改变。中国政府和人民有决心、有能力维护国家的主权和领土完整，绝不允许把台湾从祖国分裂出去，任何分裂祖国的图谋都是不能得逞的。我们寄希望于台湾人民，热忱呼吁包括台湾同胞在内的全体中国人携起手来，为早日实现祖国的完全统一而努力！历史将会永远铭记那些为实现祖国统一作出贡献的人们。

　　**八、**关于国际形势和外交工作
　　过去的五年，国际局势发生了巨大变化。在旧的世界格局瓦解、新的格局尚未形成的情况下，中国坚持独立自主的和平外交政策，经受住了国际风云变幻的严峻考验，外交工作取得重大成就。
　　我国积极改善和发展了同所有周边国家的睦邻友好关系，增进了人民之间的友好交往，促进了在平等互利基础上的经济、贸易、科技和文化合作。历史遗留下来的中国同少数邻国的边界问题，有的已经解决，有的正在谈判，有的达成了保持和平与安宁的协定。
　　我国同广大发展中国家在维护民族独立与国家主权、争取共同发展的斗争中相互同情和支持，传统友谊得到巩固，经贸关系有新的发展。我国同亚、非、拉区域性组织保持了良好的合作关系，共同维护了发展中国家的权益。
　　东欧剧变、苏联解体之后，我们尊重该地区各国人民的自主选择，在和平共处原则基础上同它们建立和发展了新的国家关系。
　　中美关系经历了严重的困难，这完全是由美国不明智的对华政策造成的。经过双方努力，现在虽然关系有所改善，但仍起伏不定。我国同西欧以及其他发达国家的关系得到全面恢复和改善，经济技术合作进入了新的发展时期。
　　我国作为联合国安理会常任理事国，积极参与国际事务和联合国各项活动，也积极参与了其他多边外交活动。中国在维护世界和平，和平解决国际争端，促进世界经济和社会发展中，发挥了重要作用。中国积极参与亚太地区经济合作，并参加了第一次亚欧会议。我国成功地承办了联合国第四次世界妇女大会。中国在国际问题上一贯坚持的公正立场和正义主张，得到越来越广泛的理解和支持。
　　当前，世界仍处在复杂而深刻的变化之中。国际形势总体趋向缓和，世界加快向多极化发展，新的格局日渐明朗。发展中国家的国际地位进一步增强。大国关系在继续调整。世界要和平，国家要稳定，经济要发展，人类要进步，已经成为当今世界的主旋律。但是，世界还充满着矛盾。霸权主义和强权政治是世界不安宁的根源。领土争端、民族矛盾、宗教纷争等因素引发的武装冲突乃至局部战争时有发生。不公正、不合理的国际政治经济秩序尚未改变，贫富差距在进一步扩大。和平与发展仍然是各国政府和人民面临的两大课题。
　　我国将继续坚定不移地奉行独立自主的和平外交政策，同世界各国发展友好合作关系，为建立公正与合理的国际政治经济新秩序而努力。在国际事务中，我们将一如既往，根据事情本身的是非曲直决定自己的立场和政策，不与任何大国或国家集团结盟，不以意识形态和社会制度为标准处理国家间关系，坚决反对任何国家以民族、宗教、人权等问题为借口干涉别国内政。
　　我国将始终不渝地执行睦邻友好政策，维护亚太地区的和平与稳定，推动亚太区域经济合作。深入发展同东盟国家的友好合作关系。维护和发展同朝鲜的传统友谊。加强同韩国的平等互利合作。本着前事不忘、后事之师的精神，发展同日本长期稳定的友好关系。继续发展同俄罗斯的友好合作，重视发挥两国在经济上的互补作用。积极发展同中亚及其他独联体国家的良好关系。继续发展同东南亚、南亚、南太平洋以及所有邻国的友好合作关系。
　　加强同广大第三世界国家的团结合作，是我国外交政策的一个基本立足点。要进一步加强同这些国家领导人的往来，加深相互理解，加强在国际问题上的磋商与合作。中国将继续向发展中国家提供力所能及的援助，积极探索优势互补的经济、贸易、科技合作的新途径。
　　中国愿意在求同存异、平等互利的基础上，继续发展同欧洲以及其他一切国家的友好合作关系，扩大经贸和科技合作。中美之间保持正常关系，不仅符合两国人民的根本利益，也有利于亚太地区乃至世界的和平与稳定。台湾问题始终是中美关系中最敏感、最重要的问题。只有严格遵守中美三个联合公报的各项原则，相互尊重，互不干涉内政，中美关系才能健康发展。
　　中国一贯反对军备竞赛，主张真正裁军。我们认为，拥有世界上最庞大核武库的国家，对军备控制负有特殊责任。中国主张全面禁止和彻底销毁核武器，主张有核国家缔结承诺不首先使用核武器、不对无核国家和地区使用或威胁使用核武器的条约。中国将继续积极参与全面禁止核试验条约的谈判，并以实际行动推动国际军备控制和裁军的进程。
　　全世界任何不带偏见的人都看得见，中国是维护世界和平与地区稳定的重要力量。中国的发展与壮大，是对世界和平与人类进步事业的贡献，绝不会对任何国家构成威胁。中国政府和人民愿意与各国政府和人民一道，为建设一个更加美好的世界作出自己的贡献！

　　**九、**努力做好1996年的工作，为“九五”创造一个良好开端
　　过去的一年，我国改革开放和经济、社会发展取得新的成就，完成了八届人大三次会议确定的任务。国内生产总值比上年增长10.2%，零售物价涨幅从21.7%回落到14.8%，既降低了物价涨幅，又保持了经济较快增长，实现了年初预定的调控目标。农业战胜了比较严重的自然灾害，粮食总产量达到4650亿公斤，增产将近200亿公斤，主要农产品产量都有不同程度的增加。工业结构继续得到调整，国有经济增长速度提高，中西部地区发展加快。进出口总额达到2800多亿美元，增长18.6%。外商直接投资实际到位370多亿美元，投资结构有所改善。国家外汇储备有较多增加，人民币汇价稳定。经济体制改革稳步推进，宏观领域的重大改革举措得到巩固和完善，建立现代企业制度的试点和城市改革试点全面展开，以养老和失业保险为主要内容的社会保障制度改革、流通体制改革，以及其他配套改革，都有新的进展。城乡居民实际收入增加，物质文化生活继续改善，储蓄存款余额增加8000多亿元。科技、教育和各项社会事业取得新的成绩，精神文明和民主法制建设得到加强。
　　1996年是“九五”计划第一年，有个好的开端非常重要。要紧紧围绕经济体制和经济增长方式的转变，安排和开展各方面的工作。今年经济增长速度的调控目标定为8%，物价上涨幅度控制在10%左右，固定资产投资率定为32%左右。要继续把抑制通货膨胀作为宏观调控的首要任务，采取有力措施控制物价上涨。在加强农业方面要作出更大的努力，力争农业有一个好收成。积极推进国有企业改革，争取在一些重点难点问题上有所突破。继续实行适度从紧的财政政策和货币政策，努力减少财政赤字，控制债务规模和货币供应总量。今年调整了进出口税收政策，要以此为契机，在促进对外经济交流与合作，提高对外开放效益和水平方面取得进步。要加强经济运行的综合协调工作，及时解决生产、建设和流通中的突出矛盾和问题。要大力整治经济秩序，严肃财经纪律，打击经济犯罪，规范市场行为，切实减轻农民负担。要加强精神文明建设，加大反腐败斗争力度，采取有力措施打击一切刑事犯罪活动，搞好社会治安综合治理。现在大政方针已定，各级政府都要认真落实，使各方面的工作都出现一个新气象。
　　各位代表！21世纪即将到来。在快要过去的20世纪，中国人民在中国共产党领导下，做了两件惊天动地的大事：前半个世纪在民族解放和人民民主革命的斗争中取得了划时代的胜利，后半个世纪在社会改革和实现繁荣富强的道路上迈出了决定性的步伐。这是中华民族历史上空前的壮举。改革开放以来三个五年计划的建设，使国家面貌发生了巨大变化。我们相信，再经过三个五年计划的建设，达到《纲要》提出的要求，我们国家的面貌就会有一个更大的变化。从现在起再经过半个世纪的努力，我们伟大的祖国，必将以现代化的崭新面貌屹立于世界。毛泽东主席说过：“人民，只有人民，才是创造世界历史的动力。”全国各族人民团结起来，在邓小平建设有中国特色社会主义的理论和党的基本路线指引下，在以江泽民同志为核心的党中央领导下，艰苦奋斗，开拓创新，一定能够实现“九五”计划和2010年远景目标，夺取社会主义现代化事业的更大胜利！

© 北大法宝：（[www.pkulaw.com](https://www.pkulaw.com/)) 专业提供法律信息、法学知识和法律软件领域各类解决方案。北大法宝为您提供丰富的参考资料，正式引用法规条文时请与标准文本核对。
欢迎查看所有[产品和服务](https://www.pkulaw.com/helps/2.html)。法宝快讯：[如何快速找到您需要的检索结果？](https://www.pkulaw.com/helps/70.html)[法宝](https://www.pkulaw.com/helps/69.html)V6有何新特色？

扫描二维码阅读原文

**原文链接：**<https://www.pkulaw.com/chl/beaee656b9b09c14bdfb.html>
